# Rapid Superficial Dehiscence After Cesarean Delivery in the Setting of Maternal Inflammation and Trauma: A Case Report

**DOI:** 10.3390/reports9030208

**Published:** 2026-07-01

**Authors:** Lexi Frankel, Courtney Marie VanderMeersch, Jeffrey Morgan Denney

**Affiliations:** Department of Obstetrics & Gynecology, Section on Maternal-Fetal Medicine, Wake Forest University School of Medicine, Medical Center Blvd, Winston Salem, NC 27157, USA; lexi.frankel@advocatehealth.org (L.F.); cvmeersch20@gmail.com (C.M.V.)

**Keywords:** wound dehiscence, absorbable skin staples, high-risk obstetric patients, case report, wound healing, high-risk pregnancy, substance use disorder in pregnancy

## Abstract

**Background and Clinical Significance:** Superficial postoperative wound dehiscence after cesarean delivery is insufficiently described in the literature, and evidence guiding management in high-risk patients remains limited. **Case Presentation:** We report a case of superficial wound dehiscence in a patient who underwent cesarean delivery following a motor vehicle accident with non-reassuring fetal heart tones and placental abruption. Her medical history included Hepatitis C infection, methadone dependence, endocarditis, and a prior episode of rapid wound dehiscence after laparoscopic surgery incisions closed with absorbable suture. **Conclusions:** Although many studies demonstrate no significant difference in dehiscence rates across closure methods, including suture, metal staples, and absorbable staples, clinicians should recognize that underlying medical conditions associated with inflammation or a history of prior wound dehiscence may increase the risk of complications when absorbable suture or absorbable staples are used. Careful assessment of patient-specific risk factors may help guide optimal closure technique in high-risk obstetric populations.

## 1. Introduction and Clinical Significance

Cesarean section is the most common surgical procedure performed in the United States, with more than one million procedures completed annually [1]. Despite its frequency, there are no standardized guidelines for optimal skin-closure techniques in cesarean delivery. Existing studies comparing closure methods, including staples, subcuticular sutures, and interrupted techniques, are limited by heterogeneous study designs, inconsistent definitions of wound complications, and variability in patient populations.

A randomized controlled trial published in 2019 evaluated skin closure in emergency cesarean delivery using staples, subcuticular Monocryl 3-0, and nylon 2-0 mattress sutures. The authors reported a higher incidence of wound complications and increased postoperative healthcare utilization in the staple group [2]. However, the generalizability of this study is limited by its modest sample size (*n* = 300). An additional limitation was the use of plain catgut for subcutaneous closure in the staple arm, a material previously associated with heightened inflammatory response and foreign-body reaction [2,3]. Furthermore, most prior studies focus on populations not at elevated risk for wound complications, limiting their applicability to medically complex obstetric patients.

Wound dehiscence is a serious postoperative complication with significant implications for healing and prognosis [4]. Superficial dehiscence involves separation of the skin and subcutaneous tissue, whereas full-thickness dehiscence extends through the fascial layer [5]. The literature has largely emphasized full-thickness dehiscence due to its high morbidity and mortality, leaving superficial dehiscence comparatively understudied. Data specifically addressing superficial wound separation after cesarean delivery are particularly sparse, despite the inherently non-sterile nature of obstetric surgery and the elevated baseline risk of wound complications in this population. Although risk models exist for predicting full-thickness dehiscence, few studies have examined risk factors for superficial separation [6]. Superficial wound dehiscence, while less catastrophic than fascial disruption, is associated with increased infection risk, prolonged hospitalization, delayed recovery, and impaired postpartum functioning [4].

Known risk factors for postoperative full-thickness abdominal dehiscence include wound infection, emergency surgery, hypoproteinemia, malignancy, anemia, and peritonitis [7,8]. Some risk factors specific to cesarean delivery have also been identified. Subcutaneous tissue thickness appears to be a key determinant of superficial dehiscence, with evidence suggesting that subcutaneous tissue approximation with absorbable suture reduces wound disruption in patients with more than 2 cm of subcutaneous tissue [9]. However, there remains a lack of data comparing absorbable suture, absorbable skin staples, and non-absorbable materials in patients with a history of wound separation or medical conditions that are predisposed to impaired healing.

A substantial gap persists in the literature regarding superficial postoperative wound dehiscence after cesarean delivery, particularly in the context of trauma or physiologic stressors that may compromise wound integrity or delay healing. This case report addresses this gap by describing a patient who developed superficial wound dehiscence following a motor vehicle accident and emergent cesarean delivery for non-reassuring fetal heart tones and placental abruption. The case highlights the need for heightened clinical awareness, improved risk stratification, and further research into optimal closure techniques. Investigations aimed at improving postoperative management strategies may prevent superficial wound complications in obstetric and other surgical patients.

## 2. Case Presentation

A 30-year-old Gravida 4 Para 1213 patient was presented to the emergency department via emergency medical services at 26 + 3 weeks of gestation following a motor vehicle collision with significant frontal impact. Her medical history included newly diagnosed Hepatitis C (viral load >3 million IU/mL, normal liver function tests), poor nutritional status, former tobacco use, BMI 27, and intravenous drug use. She had been maintained on 160 mg of methadone daily for the past year. Additional history included bacteremia-associated tricuspid valve endocarditis with known vegetation.

On arrival, she reported severe abdominal pain and vaginal bleeding. The fetal heart rate was 130 beats per minute with a reassuring pattern.

Her trauma evaluation revealed a nondisplaced transverse fracture of the right radial styloid extending into the radioscaphoid joint, a nondisplaced right ninth rib fracture, and a small chronic avulsion fracture nonunion at the medial aspect of the first metatarsal head. Given the acuity of her obstetric condition, orthopedic consultation was initially deferred.

After clearance by the trauma team, she was admitted to labor and delivery for continuous fetal monitoring. On arrival to obstetric triage, she exhibited recurrent fetal heart rate decelerations and contractions every four to five minutes. She received 12 milligrams of intramuscular betamethasone, a 4-gram magnesium sulfate bolus, and a 2-gram magnesium sulfate hourly infusion rate for fetal neuroprotection in anticipation of possible preterm delivery.

After three hours of monitoring, her vaginal bleeding worsened, and she was taken to the operating room for an emergency low-transverse cesarean section due to non-reassuring fetal heart tones. Apgar scores were 6 and 8 at one and five minutes, respectively, and the neonate weighed 0.91 kg. A large placental abruption was noted intraoperatively.

The uterine incision was repaired with 0-Monocryl in a continuous locked fashion. The fascia was closed with 0 looped PDS in a continuous fashion with the knot buried. The subcutaneous tissues were irrigated, and electrocautery was used to achieve hemostasis. Because the subcutaneous layer measured less than 2 cm, it was not closed with suture. The skin was closed with absorbable staples (Insorb). One surgeon held up both sides of the incision using two Adson forceps while the other surgeon used the Insorb device to place staples at approximately 7 mm intervals in the midline. The skin edges were slightly everted at completion of staple placement and there was no tension on the staple line. Serial postpartum laboratory studies showed no severe anemia requiring transfusion and no coagulopathy requiring product replacement.

During her postoperative course, orthopedic surgery evaluated her injuries and placed her right wrist in a splint. Orthopedic and trauma surgery recommended aggressive pulmonary toilet, incentive spirometer use, multimodal pain control, and outpatient orthopedic follow-up in one week. Cardiology reviewed her history and determined that outpatient follow-up was appropriate given her known tricuspid vegetation and tricuspid regurgitation.

**Figure 1:**
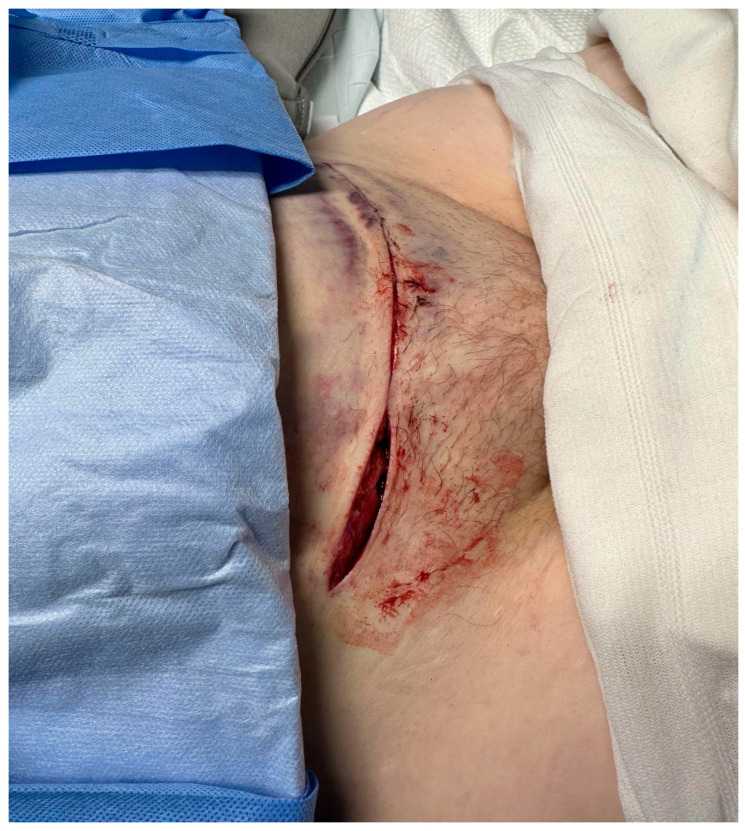
Superficial wound dehiscence of right lateral aspect of cesarean section incision on postoperative day 2.

On postoperative day 2, she was noted to have an approximately 4 cm superficial wound dehiscence on the right aspect of the skin incision (Figure 1). Upon experiencing the dehiscence, the patient indicated that subcuticular skin closure with absorbable suture led to the prior dehiscence in her cholecystectomy. She reported that she was surprised it happened with absorbable staples but postulated verbally that her “body must generate some odd response to suture or absorbable staples.” Before reapproximation, she received hydromorphone for analgesia, and the incision was cleansed with chlorhexidine. The area was anesthetized with 10 milliliters of 1 percent lidocaine.

**Figure 2:**
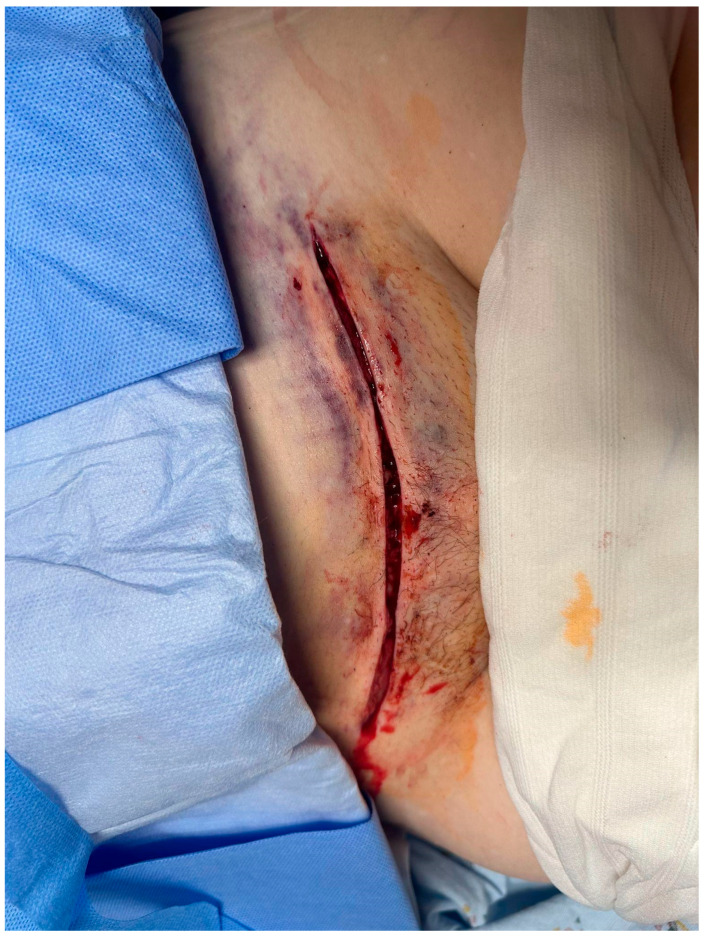
Superficial wound dehiscence of entire cesarean section incision on postoperative day 2 minutes after initial inspection of the incision, with multiple absorbable sutures partially detached under the skin.

**Figure 3:**
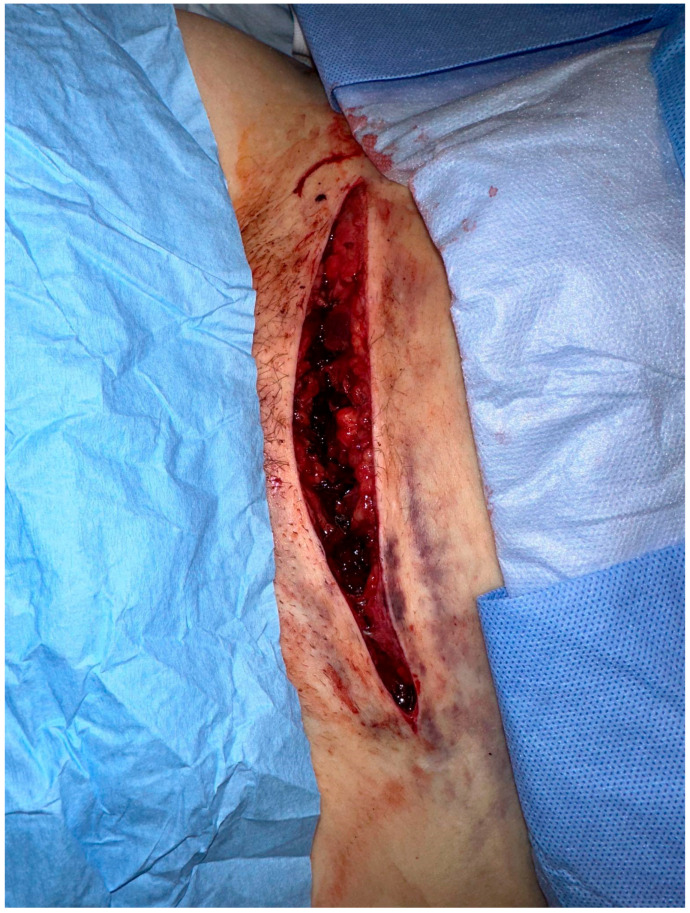
Superficial wound dehiscence of entire cesarean section incision on postoperative day 2 directly after debridement and removal of absorbable staples that were partially detached beneath the skin.

On inspection, the remainder of the incision began to separate, and the absorbable staples were nearly completely absent or absorbed (Figure 2). An additional 20 milliliters of lidocaine was administered for anesthesia. The wound was explored, and multiple absorbable sutures were removed due to detachment (Figure 3). The fascia remained intact. The skin was reapproximated with 4-0 Monocryl in a running fashion, and a dressing was applied. She tolerated the procedure well and received 2 g of cefazolin afterward. Of note, there were no clinical signs of infection at the time of debridement and wound cultures were not obtained. Specifically, an exam before closure demonstrated she was afebrile with no evidence of purulence, erythema, warmth, or induration.

Her postoperative recovery was otherwise uncomplicated, with the incision healing well and remaining clean, dry, and intact.

She met all postpartum milestones, including tolerating oral intake, ambulating, voiding, and passing flatus. She was discharged on postoperative day 5 in stable condition with strict return precautions. Follow-up referrals were arranged with Gastroenterology for Hepatitis C management, Orthopedic Surgery for her fractures, Addiction Medicine for ongoing methadone maintenance, and Cardiology for her history of endocarditis and stable tricuspid vegetation with regurgitation.

## 3. Discussion

This case highlights a rare but clinically meaningful complication associated with the use of absorbable skin staples in a high-risk obstetric patient. Although absorbable staples and sutures have demonstrated broad utility across surgical disciplines, their performance in obstetric surgery, particularly in the setting of trauma, infection, or systemic inflammatory comorbidities, remains poorly characterized [10,11]. As such, this report serves as a hypothesis-generating observation that underscores the need for prospective studies or registry-based data evaluating wound closure methods in medically complex obstetric populations; see Table 1.

The patient described here possessed multiple risk factors for impaired wound healing, any of which may have contributed to her postoperative dehiscence. Her Hepatitis C infection, with associated systemic inflammation and nutritional compromise, is known to disrupt collagen deposition and delay tissue repair. The recent motor vehicle collision introduced acute physiologic stress, triggering inflammatory and neuroendocrine cascades that can impair perfusion, increase tissue edema, and weaken wound integrity. Although she received a single dose of betamethasone for fetal lung maturity, chronic rather than single-dose corticosteroid exposure is typically associated with impaired healing, making this an unlikely contributor. In her clinical context of trauma, Hepatitis, and history of prior wound dehiscence, the evidence for benefits known and consistently demonstrated for neonatal outcomes outweigh rare but potentially subtle effects or delays on wound-healing and risk for infection with betamethasone dosed acutely, rather than chronic exposure.

Importantly, the patient reported a prior dehiscence following cholecystectomy, also involving absorbable materials. This history suggests a possible individual susceptibility to absorbable closure systems, particularly in the context of chronic viral hepatitis. In such patients, clinicians should exercise caution when selecting absorbable sutures or staples and maintain a heightened index of suspicion during postoperative wound evaluation.

Recent studies have reinforced that cesarean wound complications remain a significant source of postpartum morbidity, particularly among patients with medical comorbidities or physiologic stressors that impair tissue repair [12]. Emerging data suggest that absorbable skin closure systems, while effective in low-risk populations, may be associated with higher rates of superficial separation in patients with inflammatory or infectious conditions, although evidence remains limited and inconsistent across studies [13]. Contemporary reviews have emphasized the importance of individualized postoperative wound management, noting that early identification of high-risk patients and proactive surveillance can reduce the likelihood of progression from superficial separation to deeper infection or fascial involvement [14,15].

The newer literature from 2024 to 2026 further supports the need for tailored closure strategies. A 2024 multicenter analysis demonstrated that patients with chronic inflammatory disease or recent physiologic stressors had significantly higher odds of superficial wound separation regardless of closure material, suggesting that patient level factors may outweigh the effect of closure technique alone [16]. A 2025 systematic review evaluating absorbable dermal closure systems reported increased rates of early superficial separation in patients with infectious comorbidities, although overall healing outcomes remained comparable when appropriate postoperative surveillance was implemented [17]. Additionally, a 2026 cohort study examining postoperative wound management in high-risk obstetric patients found that structured follow-up protocols reduced the incidence of delayed wound complications by improving early detection and intervention [18].

Adjunctive therapies such as negative pressure wound therapy and Z-plasty have demonstrated benefits in non-obstetric surgical cohorts, including enhanced granulation tissue formation, reduced bacterial burden, and accelerated closure [4]. However, these modalities remain insufficiently studied in post cesarean or obstetric trauma patients. This gap highlights the need for further research in obstetric populations to determine whether these strategies are similarly effective following cesarean delivery, particularly in patients at elevated risk [4].

Future studies are needed to evaluate risk factors for wound complications in cesarean incisions, especially among patients with infectious disease, recent trauma, or a history of wound-healing difficulties. Such research would help clarify whether absorbable materials confer additional risk in these populations and would support evidence-based recommendations for closure technique selection.

## 4. Conclusions

This case report is inherently limited by its single-patient design, which restricts generalizability. Nevertheless, the observed wound dehiscence in the setting of trauma and maternal inflammatory comorbidities is consistent with prior reports describing dehiscence among patients with significant systemic illness or physiologic stressors. What remains unclear, both in this case and in the previously published literature, is whether the disruption of the wound was accelerated by the patient’s underlying Hepatitis C infection, the physiologic consequences of blunt abdominal trauma, or a combination of these factors.

Although absorbable staples have demonstrated safety and efficacy in uncomplicated cesarean deliveries, this case raises important questions about their use in patients with multiple concurrent risk factors for impaired healing or postoperative complications such as infection, seroma, or abscess. Because this report describes a single patient, causation cannot be inferred. It is also possible that a non-absorbable closure might have failed under similar conditions.

As outlined in the introduction, multiple cohort studies have shown no significant difference in dehiscence rates when comparing metal staples, traditional sutures, and absorbable staple systems for cesarean skin closure. Taken together with our findings, this suggests that the choice of closure material alone is unlikely to be the dominant determinant of wound integrity. Instead, a patient’s underlying medical conditions, including chronic or acute inflammation, edema, impaired collagen deposition, malnutrition, or a history of prior wound-healing complications, may exert a more substantial influence, particularly when several such factors are present simultaneously.

As with many conditions in obstetrics, history is a strong predictor of recurrence. Trauma, physiologic stress, infection, malnutrition, and inflammatory states all contribute meaningfully to wound-healing risk. To better guide clinical decision-making, further research is needed in the form of case–control studies, prospective cohorts, or randomized controlled trials evaluating closure techniques in high-risk obstetric populations. These groups are inherently heterogeneous and would require large sample sizes, which may explain the current lack of robust data.

In the interim, clinicians should evaluate the entire clinical context, including both historical and acute factors, when selecting a wound-closure method for cesarean delivery. For patients with multiple risk factors, individualized assessment may help optimize healing and guide the choice of closure material.

## Figures and Tables

**Table 1:** Patient risk factors and associated pathophysiology of impaired healing.

Risk Factor	Potential Mechanism of Impaired Healing
Hepatitis C infection	Chronic inflammation, nutritional compromise, impaired collagen deposition
Recent blunt trauma (MVA)	Tissue edema, inflammatory surge, impaired perfusion, physiologic stress response
Poor nutritional status	Reduced substrate availability for collagen synthesis and tissue repair
History of bacteremia and tricuspid endocarditis	Systemic inflammation, potential immune dysregulation
Intravenous drug use	Vascular injury, immune compromise, nutritional deficits
Former tobacco use	Microvascular impairment, reduced oxygen delivery
Prior wound dehiscence with absorbable materials	Suggests individual susceptibility or impaired tissue response
BMI 27	Mildly increased tension on wound edges

## Data Availability

The original contributions presented in this study are included in the article. Further inquiries can be directed to the corresponding author.

## Abbreviations

The following abbreviations are used in this manuscript:

CS	Cesarean section
MVA	Motor vehicle accident
